# Demyristoylation of the Cytoplasmic Redox Protein Trx-h2 Is Critical for Inducing a Rapid Cold Stress Response in Plants

**DOI:** 10.3390/antiox10081287

**Published:** 2021-08-13

**Authors:** Eun Seon Lee, Joung Hun Park, Seong Dong Wi, Ho Byoung Chae, Seol Ki Paeng, Su Bin Bae, Kieu Anh Thi Phan, Min Gab Kim, Sang-Soo Kwak, Woe-Yeon Kim, Dae-Jin Yun, Sang Yeol Lee

**Affiliations:** 1 Division of Applied Life Science (BK21+) and PMBBRC, Gyeongsang National University, Jinju 52828, Korea; dmstjsl88@hanmail.net (E.S.L.); jazzc@nate.com (J.H.P.); wsd3377@gmail.com (S.D.W.); truedaisy@hanmail.net (H.B.C.); skpaeng@gmail.com (S.K.P.); subin7760@hanmail.net (S.B.B.); phanthikieuanh95@gmail.com (K.A.T.P.); kim1312@gnu.ac.kr (W.-Y.K.); 2 College of Pharmacy, Gyeongsang National University, Jinju 52828, Korea; mgk1284@gnu.ac.kr; 3 Plant Systems Engineering Research Center, KRIBB, Daejeon 34141, Korea; sskwak@kribb.re.kr; 4 Department of Biomedical Science & Engineering, Konkuk University, Seoul 05029, Korea; djyun@konkuk.ac.kr

**Keywords:** thioredoxin h2, myristoylation/demyristoylation, nuclear translocation, C-repeat binding factors (CBFs), cold/freezing stress, Trx-h2(G/A) point mutation variant, transgenic Arabidopsis

## Abstract

In Arabidopsis, the cytosolic redox protein thioredoxin h2 (Trx-h2) is anchored to the cytoplasmic endomembrane through the myristoylated second glycine residue (Gly^2^). However, under cold stress, the cytosolic Trx-h2 is rapidly translocated to the nucleus, where it interacts with and reduces the cold-responsive C-repeat-binding factors (CBFs), thus activating *cold-responsive (COR)* genes. In this study, we investigated the significance of fatty acid modification of Trx-h2 under cold conditions by generating transgenic Arabidopsis lines in the *trx-h2* mutant background, overexpressing *Trx-h2* (Trx-h2^OE^/*trx-h2*) and its point mutation variant *Trx-h2(G/A)* [Trx-h2(G/A)^OE^/*trx-h2*], in which the Gly^2^ was replaced by alanine (Ala). Due to the lack of Gly^2^, Trx-h2(G/A) was incapable of myristoylation, and a part of Trx-h2(G/A) localized to the nucleus even under warm temperature. As no time is spent on the demyristoylation and subsequent nuclear translocation of Trx-h2(G/A) under a cold snap, the ability of Trx-h2(G/A) to protect plants from cold stress was greater than that of Trx-h2. Additionally, *COR* genes were up-regulated earlier in Trx-h2(G/A)2^OE^/*trx-h2* plants than in Trx-h2^OE^/*trx-h2* plants under cold stress. Consequently, Trx-h2(G/A)2^OE^/*trx-h2* plants showed greater cold tolerance than Col-0 (wild type) and Trx-h2^OE^/*trx-h2* plants. Overall, our results clearly demonstrate the significance of the demyristoylation of Trx-h2 in enhancing plant cold/freezing tolerance.

## 1. Introduction

Plants, being sessile organisms, are routinely exposed to diverse environmental stresses, such as heat, cold, drought, salinity, heavy metal toxicity, osmotic stress, and pathogen attack [1]. In response to these biotic and abiotic stresses, plants produce various kinds of reactive oxygen species (ROS) including hydroxyl radical, superoxide anion, and hydrogen peroxide, which play dual roles by acting as intracellular signaling molecules (at optimal concentrations) and as cytotoxic compounds (at high concentrations) [2,3,4,5]. Increased accumulation of ROS under stress conditions leads to the denaturation of crucial intracellular macromolecules, followed by cell death, whereas low ROS level causes growth arrest and dwarfism in plants [6,7]. Thus, maintenance of ROS homeostasis is critical for balancing plant growth and development with stress tolerance [8,9]. To maintain ROS homeostasis, plants employ many small antioxidant molecules and a number of antioxidant enzymes belonging to large protein families, such as catalases, peroxidases, thioredoxins (Trxs), glutaredoxins, peroxiredoxins and protein disulfide reductases [10,11,12,13]. The number of antioxidant proteins found in plants is significantly higher than that in mammals and microorganisms.

The Trx family proteins, Trx-f, Trx-m, Trx-h, Trx-o, Trx-x, and Trx-y, regulate diverse cellular processes, such as cell division, apoptosis, seed germination, photosynthesis, and stress resistance, by controlling the redox status of target signaling molecules [14,15]. Among these, the cytosolic isoforms of Trx-hs regulate innumerable cellular signaling processes, such as heat and cold shock resistance, plant immunity, vesicle trafficking, and plant growth [16,17,18]. The N-terminal amino acids of Trx-hs undergo various covalent modifications; for example, myristoylation of the N-terminal glycine (Gly) residue, and prenylation or palmitoylation of the cysteine (Cys) residue [19]. Similar to various post-translational modifications, including phosphorylation, ubiquitination, disulfide bond formation and SUMOylation [20,21,22], the lipid modification of Trx-hs affects their stability, subcellular localization, membrane trafficking, secretion, interaction with target molecules, and rapid response to external stresses [23,24,25,26]. In *Arabidopsis thaliana*, the cytosolic Trx-h2 isoform was recently shown to protect plants from freezing stress via its cold-induced nuclear translocation and interaction with key cold-responsive transcription factors, namely, C-repeat binding factors (CBFs) [27]. In the nucleus, Trx-h2 reduces the oxidized (inactive) CBF oligomers and dissociates their protein structure to produce CBF monomers. The reduced (active) CBF monomers activate the expression of various *cold-responsive (COR)* genes, which protect plants from freezing stress by enhancing plant cold tolerance. We previously showed that the myristyl group, covalently attached to Gly^2^ of Trx-h2 at warm temperature, was rapidly cleaved by cold shock, which allowed the nuclear translocation of Trx-h2 through the nuclear localization signal (NLS) located at its C-terminus. This suggests that demyristoylation at Gly^2^ is essential for Trx-h2-mediated regulation of the cold stress response in plants.

In this study, we investigated the physiological significance of myristoylation at Gly^2^ of Trx-h2. We overexpressed *Trx-h2* and its point mutation variant *Trx-h2(G/A)* in the *trx-h2* mutant background to generate Trx-h2^OE^/*trx-h2* and Trx-h2(G/A)^OE^/*trx-h2* transgenic lines, respectively. As Gly^2^ was replaced by alanine (Ala) in Trx-h2(G/A), the mutant protein did not show myristoylation. The subcellular localization of Trx-h2, structural changes in CBFs, expression level of *COR* genes, and resistance to cold and freezing stresses were compared among the Trx-h2^OE^/*trx-h2*, Trx-h2(G/A)^OE^/*trx-h2*, *trx-h2* mutant, and Col-0 (wild type) plants. From the studies, we demonstrated that Trx-h2(G/A)2^OE^/*trx-h2* plants showed greater cold tolerance than Col-0 and Trx-h2^OE^/*trx-h2* plants, suggesting that the demyristoylation of Trx-h2 is critical for inducing a rapid cold stress response in plants.

## 2. Materials and Methods

### 2.1. Plant Materials and Growth Conditions

*Arabidopsis thaliana* ecotype Columbia (Col-0; wild type (WT)) and T-DNA insertion mutant *trx-h2* (SALK_079507) were used in this study. Seeds of both genotypes were obtained from the Arabidopsis Biological Resource Center (ABRC; Ohio State University, Columbus, OH, USA). Plants were grown in a plant culture chamber maintained at 22 °C, 70% relative humidity, and a 16 h light/8 h dark photoperiod.

### 2.2. Plasmid Construction and Plant Transformation

A full-length *Trx-h2* coding sequence was amplified from Arabidopsis cDNA library by PCR using Trx-h2_F and Trx-h2_R primers (Table S1). The PCR product was cloned into the pGEM-T Easy vector (Promega Co., Madison, WI, USA). The resulting pGEM-T:*Trx-h2* construct was used to synthesize *Trx-h2(G/A)* by PCR-based site-directed mutagenesis using Trx-h2(G/A)_F and Trx-h2_R primers. The PCR product was cloned into the pGEM-T Easy vector to generate the final construct, designated as pGEM-T:*Trx-h2(G/A)*. Primers used for cloning are listed in Table S1. *Trx-h2* and Trx-h2(G/A) were fused with the yellow fluorescent protein (YFP) gene to generate *Trx-h2-YFP* and *Trx-h2(G/A)-YFP*. To produce Trx-h2-YFP^OE^/*trx-h2* and Trx-h2(G/A)-YFP^OE^/*trx-h2* transgenic plants, pGEM-T:*Trx-h2-YFP* and pGEM-T:*Trx-h2(G/A)-YFP* constructs were cloned into the pCAMBIA1300 binary vector. The resulting plasmids were introduced into *Agrobacterium tumefaciens* strain GV3101, and the resulting cultures were used to transform *trx-h2* plants using the floral dip method [28]. Homozygous T3 lines were used for subsequent experiments.

### 2.3. Detection of the Myristoylation of Trx-h2 In Vivo

The presence of the C-14 myristyl group in Trx-h2 was detected as described previously [27]. Briefly, 2-week-old Trx-h2-YFP^OE^*/trx-h2* or Trx-h2(G/A)-YFP^OE^*/trx-h2* seedlings were vacuum-infiltrated in 40 μM azidomyristate (myristic acid, azide; Life Technologies, Camarillo, CA, USA) and incubated at 22 °C for 1 day. Subsequently, the seedlings were ground in liquid nitrogen, and total proteins were extracted using the immunoprecipitation (IP) buffer containing 50 mM Tris-HCl (pH 7.5), 150 mM NaCl, 1 mM EDTA, 0.5% NP-40, 1 mM phenylmethylsulfonyl fluoride (PMSF), and protease inhibitor cocktail. After centrifugation, 250 μM phosphine-PEG_3_-biotin was added to the supernatant (250 μg protein) and incubated at 37 °C for 2 h. The biotinylated proteins were immunoprecipitated using protein-A agarose beads conjugated to anti-GFP antibody (Thermo Fisher Scientific, Rockford, IL, USA), and then separated by sodium dodecyl sulfate-polyacrylamide gel electrophoresis (SDS-PAGE). Biotinylated azidomyristoylated Trx-h2-YFP was detected by Western blotting using anti-biotin antibody (Abcam, Cambridge, MA, USA) and anti-GFP antibody.

### 2.4. Subcellular Fractionation of Nuclear and Non-Nuclear Proteins

Nuclear proteins were isolated from 2-week-old cold (4 °C)-treated and untreated Trx-h2-YFP^OE^/*trx-h2* and Trx-h2(G/A)-YFP^OE^/*trx-h2* plants using the CelLytic PN Extraction Kit (Sigma-Aldrich, St. Louis, MO, USA), according to the manufacturer’s instructions. Briefly, plant tissues were frozen, ground in liquid nitrogen, and mixed with 1× Nuclei Isolation Buffer (NIB). Samples were centrifuged, and the supernatant was separated from the pellet. The pellet containing nuclear proteins was mixed with 1× NIBA buffer containing 10% Triton X-100. The lysates were overlayed on top of a 1.5 M sucrose cushion. After centrifugation, the pellet was resuspended in nuclear extraction buffer and used as the nuclear fraction. The nuclear fraction was confirmed by Western blotting with anti-histone H3 antibody (Abcam) and anti-PEPC antibody (Agrisera, Vännäs, Sweden) as nuclear and non-nuclear markers, respectively.

### 2.5. Co-immunoprecipitation (Co-IP) Assay

After grinding the frozen samples of 2-week-old Trx-h2-YFP^OE^/*trx-h2* and Trx-h2(G/A)-YFP^OE^/*trx-h2* plants, total proteins were extracted using the IP buffer. Total proteins were incubated with protein-A agarose beads and anti-GFP antibody at 4 °C, and then washed three times with IP buffer. Trx-h2-YFP and Trx-h2(G/A)-YFP proteins were eluted off the beads by heating, and then separated by SDS-PAGE. CBF and Trx-h2 proteins were detected by Western blotting with anti-CBF and anti-Trx-h2 antibodies, respectively.

### 2.6. Bimolecular Fluorescence Complementation (BiFC) Assay

Interaction of CBF1 with Trx-h2 and Trx-h2(G/A) was analyzed using the BiFC assay. *Trx-h2*, *Trx-h2(G/A)*, and *CBF1* were cloned into the pDONR221 binary vector to generate pDONR221:*Trx-h2*, pDONR221:*Trx-h2(G/A)*, and pDONR221:*CBF1* plasmids, respectively, using sequence-specific primers (Table S1)*. Trx-h2* was fused with the N-terminal fragment of the *YFP* gene (YN) to generate *Trx-h2-YN*, and *CBF1* was fused with the C-terminal fragment of *YFP* (YC) to generate *YC-CBF1.* The plasmids were introduced into *A. tumefaciens* strain GV3101, and the transformed Agrobacterium cells were used to infiltrate to the leaves of *Nicotiana benthamiana* plants. After agroinfiltration, the plants were incubated in a plant culture chamber for 2 days and then subjected to cold stress at 4 °C. Fluorescence signals were analyzed under a confocal microscope.

### 2.7. Detection of the Structural Switching of CBF1 In Vivo

Two-week-old Col-0, *trx-h2*, Trx-h2-YFP^OE^/*trx-h2*, and Trx-h2(G/A)-YFP^OE^/*trx-h2* plants were incubated at 4 °C for 3 and 6 h. After the cold treatment, plants were frozen and ground in liquid nitrogen. Total proteins were extracted from the ground tissues, and then separated by SDS-PAGE using non-reducing and reducing gels. The structural changes in CBF1 were detected by Western blotting using anti-CBF antibody.

### 2.8. Purification of Trx-h2, Trx-h2(G/A), and Maltose Binding Protein (MBP)-CBF1 Recombinant Proteins

Full-length coding sequences of *Trx-h2* and *Trx-h2(G/A)* were cloned into the pET28a vector, and *CBF1* genes was cloned into the pMAL1119 vector. Each plasmid was introduced into *Escherichia coli* BL21 (DE3) pLysS cells, and the transformed cells were cultured in Luria–Bertani (LB) medium at 37 °C until reaching an optimal density of 0.5 at 600 nm. Protein expression was induced by the addition of isopropyl-β-D-thiogalactopyranoside (IPTG) to the growth medium, and the culture was grown at 30 °C. After centrifugation, cells were resuspended in phosphate-buffered saline (PBS; 1.8 mM KH_2_PO_4_ (pH 8.0), 140 mM NaCl, 2.7 mM KCl, and 10 mM Na_2_HPO_4_). After sonication, Trx-h2 and Trx-h2(G/A) proteins were purified from the disrupted cells using Ni-NTA agarose gel, while CBF1 was purified using amylose resin. MBP-tagged CBF1 (MBP-CBF1) was eluted with 10 mM maltose, and Trx-h2 and Trx-h2(G/A) were eluted by thrombin cleavage. The eluted proteins were dialyzed with 20 mM HEPES-NaOH (pH 8.0) and used for further experimentation.

### 2.9. Electrophoretic Mobility Shift Assay (EMSA)

Biotin-labeled oligonucleotide probes (Table S1) were treated with an EMSA Kit (Thermo Fisher Scientific), as described previously [29]. The recombinant MBP-CBF1 protein was reacted with an in vivo electron transport Trx system containing NADPH, Trx reductase, and Trx-h2 or Trx-h2(G/A). Additionally, the reaction mixture was incubated with biotin-labeled probe in a solution containing Poly (dI-dC) binding buffer. The reaction products were separated on polyacrylamide gels. The DNA–protein complexes were transferred onto the Hybond-N membrane and detected by Western blot using anti-biotin antibody (Abcam).

### 2.10. CBF1 Transactivation Assay

Transactivation of CBF1 was analyzed as described previously [29]. A reporter construct (*P_COR15a_:LUC*) was co-transformed with one of the three effector constructs (*P_35S_:CBF1*, *P_35S_:Trx-h2*, and *P_35S_:Trx-h2(G/A)*) and an internal control construct (*P_35S_:GUS*) into *A. tumefaciens* strain GV3101, and the transformed cells were infiltrated into *N. benthamiana* leaves. After 2 days of incubation in the plant culture chamber, plants were treated with cold stress at 4 °C. Then, total proteins were extracted from the ground leaf tissues using IP buffer. The protein extract was mixed with the β-glucuronidase (GUS)/luciferase (LUC) enzyme solution containing 50 mM Na_2_PO_4_ (pH 7.0), 10 mM EDTA, 10 mM β-mercaptoethanol, and 0.1% Triton X-100. Then, the LUC substrate (20 mM Tricine, 2.7 mM MgSO_4_, 30 mM DTT, 1 mM luciferin, and 0.5 mM ATP) was added to the sample, and the mixture was incubated for 10 min. LUC activity was measured with a luminometer (Promega). To analyze GUS activity, total protein extracts were mixed with the GUS/LUC substrate solution containing 16.7% methanol and 1.1 mM 4-methylumbelliferyl-β-D-glucuronide hydrate (MUG) in the GUS/LUC reaction buffer, and the sample was incubated for 10 min (Sigma-Aldrich). The reaction was stopped by the addition of 130 mM Na_2_CO_3_, and MUG fluorescence was measured with a spectrofluorometer at excitation and emission wavelengths of 364 and 447 nm, respectively. LUC activity was normalized relative to GUS activity.

### 2.11. RNA Isolation and Quantitative Real-time PCR (qRT-PCR)

Total RNA was isolated from cold-treated and untreated Col-0, *trx-h2*, Trx-h2-YFP^OE^/*trx-h2*, and Trx-h2(G/A)-YFP^OE^/*trx-h2* plants using RNA Purification Kit (Macherey-Nagel, Düren, Germany). Then, cDNA was synthesized from the isolated total RNA using a cDNA synthesis kit (Thermo Fisher Scientific), and qRT-PCR was performed using cDNA as a template under the following conditions: 5 min incubation at 95 °C, followed by 25 cycles of 30 s at 95 °C, 30 s at 56 °C, and 1 min at 72 °C. *Actin2* (*ACT2*) and *Ubiquitin10* (*UBQ10*) genes were used as internal controls. Three biological replicates were performed using gene-specific primers (Table S1).

### 2.12. Freezing Tolerance Assay

Soil-grown 18-day-old Arabidopsis plants were used for the freezing tolerance assay. In the non-acclimated (NA) treatment, plants were subjected directly to freezing stress, whereas in the cold-acclimated (CA) treatment, plants were acclimated to a low temperature (4 °C) for 5 days before being subjected to freezing stress. The freezing stress treatment was conducted using the RuMED4001 freezing chamber (RuMED4001, Stuttgart, Germany). The chamber was cooled down to the target freezing temperature, which was held constant for 2 h. After the freezing stress treatment, plants were incubated at 22 °C for 5 days, as described previously [30,31]. Freezing stress tolerance of transgenic and mutant plants was examined by analyzing the morphological phenotypes, survival rate, and electrolyte leakage (%) in comparison with those of Col-0 plants.

### 2.13. Statistical Analysis

The statistical significance of the survival rate and ion leakage data was examined using Student’s *t*-test.

## 3. Results

### 3.1. Myristoylation of Arabidopsis Trx-h2 Is Dependent on the Second Amino Acid of Trx-h2, Gly^2^

**Figure 1:**
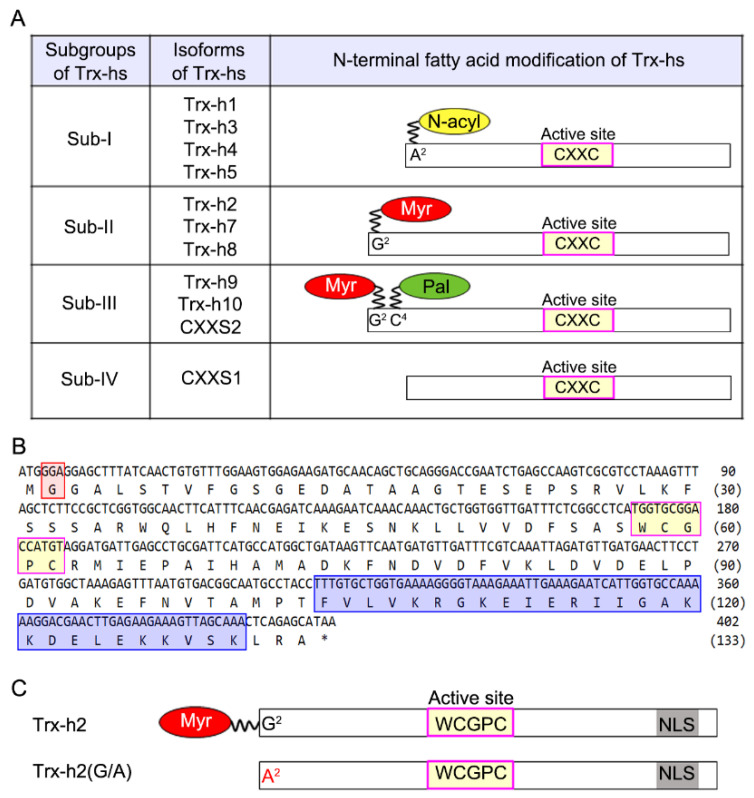
Schematic representation of the four Arabidopsis Trx-h subgroups, Trx-h2 amino acid sequence, and Trx-h2 and Trx-h2(G/A) DNA constructs. (**A**) Classification of Trx-h subgroups in Arabidopsis. The Trx-h isoforms are categorized into four subgroups, depending on the fatty acid modification of N-terminal amino acids. Critical amino acids for fatty acid modifications include Ala^2^ (A^2^), GLy^2^ (G^2^), and Cys^4^ (C^4^). N-α-acetylation, myristoylation, and palmitoylation are indicated by yellow, red, and green circles, respectively. The active site of Trx-hs containing the CXXC motif is indicated by the magenta box. (**B**) Amino acid sequence of Trx-h2. The second Gly (Gly^2^) residue of Trx-h2, involved in myristoylation, is indicated by a red box, and the WCGPC motif and bipartite NLS are indicted by yellow and blue boxes, respectively. (**C**) Schematic of the protein constructs of Trx-h2 and Trx-h2(G/A). Gly^2^ residue in Trx-h2 is indicated in black font, while its substitution, Ala^2^, in Trx-h2(G/A), is indicated in red font. The NLS is represented by a gray box.

To analyze the physiological significance of Gly^2^ in the myristoylation of Arabidopsis Trx-h2, we aligned the amino acid sequences of 11 Trx-h isoforms and investigated their sequence characteristics (Figure 1). Based on their N-terminal amino acid sequence, the Arabidopsis Trx-hs were classified into four subgroups (Sub-I to Sub-IV) [32,33,34]. All 11 Trx-hs contained two conserved Cys residues at the active site. Compared with Sub-I Trx-hs, the Sub-II and Sub-III Trx-hs harbored an extension of approximately 20 additional amino acid residues at the N-terminus. This ~20-amino acid extension is presumed to regulate the subcellular localization, protein stability, and interaction specificity of Trx-hs. The second Ala (Ala^2^) residue of Sub-I Trx-hs is responsible for protein acetylation [29], while the second Gly (Gly^2^) and fourth Cys (Cys^4^) residues of Sub-II and Sub-III Trx-hs are predicted to undergo myristoylation and palmitoylation, respectively (Figure 1A). By contrast, the monothiol active site Cys residue in the CXXS motif of Sub-IV Trx-hs is not predicted to undergo any post-translational modification.

Among the Sub-II Trx-hs, Trx-h2, which contains the NLS at its C-terminus, is predicted to be myristoylated at the Gly^2^ residue (Figure 1B) and has been shown to participate in freezing stress tolerance in plants. Considering the importance of the Trx-h2–CBF–COR signaling cascade in plants, we investigated the role of myristoylation at the Gly^2^ residue of Trx-h2 in the rapid response of plants to cold shock. To elucidate the effect of myristoylation of Trx-h2 on the cold stress response of plants, we obtained Arabidopsis *trx-h2* knockout mutant from ABRC (accession #: 079507) and prepared the Trx-h2(G/A) point mutation variant of Trx-h2, in which the Gly^2^ was replaced with Ala and was incapable of myristoylation (Figure 1C). Next, we generated transgenic Arabidopsis lines overexpressing YFP fusions of Trx-h2 and Trx-h2(G/A) in the *trx-h2* mutant background (Trx-h2-YFP^OE^/*trx-h2* and Trx-h2(G/A)-YFP^OE^/*trx-h2*, respectively) (Figure S1A,B). The knockout and overexpression (OE) lines were confirmed by Western blot analysis (Figure S1C). To verify the presence of the myristyl moiety of Trx-h2 in Trx-h2-YFP^OE^/*trx-h2* and Trx-h2(G/A)-YFP^OE^/*trx-h2* plants, 2-week-old plants were treated with azidomyristate, as described previously [35]. After the reaction, biotin-labeled polyethylene glycol (PEG_3_) was incubated with the protein extracts of Trx-h2-YFP^OE^/*trx-h2* and Trx-h2(G/A)-YFP^OE^/*trx-h2* plants. The biotin-labeled azidomyristoylated Trx-h2-YFP was immunoprecipitated using anti-GFP antibody, and the myristyl moiety was detected with anti-biotin antibody. The biotinylated myristate was detected only in the protein extract of Trx-h2-YFP^OE^/*trx-h2* plants, but not in that of Trx-h2(G/A)-YFP^OE^/*trx-h2* plants, as expected (Figure S1D). This result clearly demonstrates that Gly^2^ is critical for the myristoylation of Trx-h2 in plants.

### 3.2. Kinetics of the Nuclear Translocation of Trx-h2 and Trx-h2(G/A) in Plants

**Figure 2:**
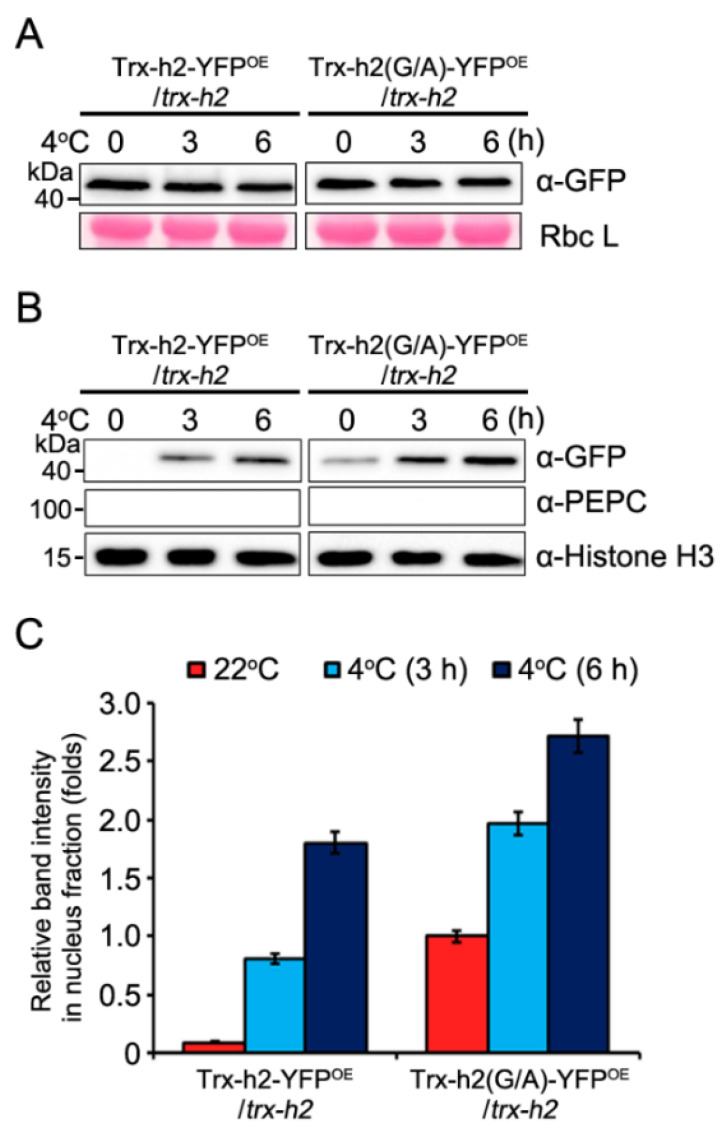
Comparison of the subcellular distribution of Trx-h2 and Trx-h2(G/A) in Trx-h2-YFP^OE^/*trx-h2* and Trx-h2(G/A)-YFP^OE^/*trx-h2* plants at different temperatures. (**A**) Abundance of Trx-h2 and Trx-h2(G/A) proteins in transgenic plants exposed to cold stress at 4 °C for the indicated duration. Trx-h2 and Trx-h2(G/A) levels were analyzed by Western blotting with anti-GFP antibody. RbcL stained with Ponceau S was used as a loading control. (**B**) Comparison of the nuclear localization of Trx-h2 and Trx-h2(G/A) in transgenic plants at 22 °C and 4 °C. Nuclear protein fractions were extracted from the plants, and Trx-h2 and Trx-h2(G/A) were detected by Western blotting using anti-GFP antibody. Anti-PEPC and anti-H3 antibodies were used as non-nuclear and nuclear markers, respectively. (**C**) Quantitative analysis of GFP band intensities in panel (**B**) measured with a densitometer and quantified by the ImageJ software. Trx-h2 and Trx-h2(G/A) protein levels were normalized relative to histone H3 levels. The protein band intensity of Trx-h2 and Trx-h2(G/A) in upper lane of panel (**B**) was normalized to the value of Trx-h2(G/A) obtained from the untreated sample (the first lane in the upper panel of Trx-h2(G/A)). Data represent mean ± standard error of mean (s.e.m.; *n* = 3 independent biological replicates).

As cold induces the translocation of Trx-h2 from the nucleus to the cytoplasm, we compared the speed of nuclear translocation of Trx-h2 and Trx-h2(G/A) in two transgenic Arabidopsis lines, Trx-h2-YFP^OE^/*trx-h2* and Trx-h2(G/A)-YFP^OE^/*trx-h2*, at 22 °C and 4 °C. Western blotting analysis showed that the levels of Trx-h2 and Trx-h2(G/A) proteins were similar under normal and cold conditions (Figure 2A). From the transgenic plants, we extracted the nuclear proteins and kinetically compared the nuclear distribution of Trx-h2 and Trx-h2(G/A). The results showed a different rate of translocation of the two proteins at 22 °C. While a negligible amount of Trx-h2 was detected in the nuclear fraction isolated from Trx-h2-YFP^OE^/*trx-h2* plants at 22 °C, a critical level of Trx-h2(G/A) was detected in the nuclear protein fraction of Trx-h2(G/A)-YFP^OE^/*trx-h2* plants at 22 °C (Figure 2B, the first lane of upper panel). These results confirmed that the myristyl group covalently linked to the Gly^2^ residue of Trx-h2 was trapped at the cytosolic endomembrane compartments, whereas a part of Trx-h2(G/A), which is incapable of myristoylation, translocated to the nucleus under normal conditions. Additionally, under cold conditions, the nuclear translocation rate of Trx-h2(G/A) was faster and higher than that of Trx-h2, resulting in greater accumulation of Trx-h2(G/A) in the nucleus compared with Trx-h2, as analyzed by Western blotting (Figure 2B). After measuring the intensities of the two nuclear proteins by densitometer, relative levels of the two proteins were normalized to the value of Trx-h2(G/A) at 22 °C (Figure 2C). Based on these results, we speculate that the demyristoylation reaction of Trx-h2 is the rate-limiting step in cold-induced nuclear translocation of Trx-h2.

### 3.3. Effect of Myristoylation on the Interaction of Trx-h2 with CBFs

**Figure 3:**
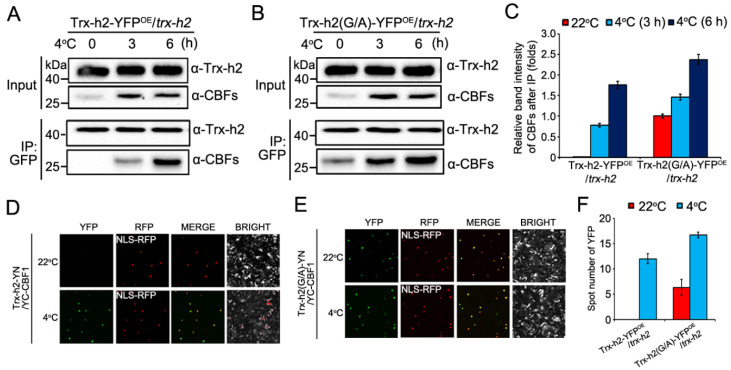
Comparison of the kinetics of nuclear translocation between Trx-h2 and Trx-h2(G/A), and of their interaction with CBFs at different temperatures. (**A**,**B**) Co-IP assay of Trx-h2-YFP and Trx-h2-YFP(G/A) proteins extracted from Trx-h2-YFP^OE^/*trx-h2* (**A**) and Trx-h2(G/A)-YFP^OE^/*trx-h2* (**B**) plants treated with cold stress at 4 °C for the indicated duration. The immunoprecipitates were separated by SDS-PAGE and analyzed by Western blotting. (**C**) Quantitative analysis of the GFP band intensities in the bottom panels of (**A**) and (**B**) measured by densitometry and quantified by the ImageJ software. Interaction band intensity between Trx-h2 or Trx-h2(G/A) with CBFs was normalized by the immunoprecipitated level of Trx-h2(G/A) (in the first lane, bottom of panel (**B**)). The Trx-h2(G/A)–CBF interaction intensity at 22 °C, shown in the first lane in bottom panel (**B**), was set to 1. (**D**,**E**) BiFC assay performed by co-expressing *Trx-h2-YN* and *YC-CBF1* (**D**) or *Trx-h2(G/A)-YN* and *YC-CBF1* (**E**) in *N. benthamiana* leaves. Samples were incubated at 4 °C and 22 °C for 6 h. YFP signals analyzed by confocal microscopy were merged with the NLS-RFP signal (nuclear marker). (**F**) The YFP fluorescent-spots in panels (**D**,**E**) were counted and compared. Data represent mean ± s.e.m. (*n* = 3 biologically independent samples).

Myristoylation of intracellular proteins plays an important role in a number of physiological processes in plants [36,37]. Thus, we investigated the role of Gly^2^ in the interaction between Trx-h2 and CBFs at warm and cold temperatures. Using the Trx-h2-YFP^OE^/*trx-h2* and Trx-h2(G/A)-YFP^OE^/*trx-h2* plants incubated at 22 °C and 4 °C, the interaction of Trx-h2 and Trx-h2(G/A) with CBFs was examined by co-IP and BiFC assays. To carry out the co-IP assay, Trx-h2 and Trx-h2(G/A) proteins were immunoprecipitated with anti-GFP antibody, and CBFs were detected using an anti-CBF antibody. Trx-h2 showed interaction with CBFs only at 4 °C, and the strength of this interaction increased with the duration of the cold treatment (Figure 3A). By contrast, Trx-h2(G/A) interacted with CBFs at both 22 °C and 4 °C, and its interaction with CBFs was stronger than that of Trx-h2 at 4 °C, confirming that the myristyl group of Trx-h2 was cleaved by cold, after which Trx-h2 translocated to the nucleus and interacted with CBFs (Figure 3B). The strength of the interaction of Trx-h2 and Trx-h2(G/A) with CBFs was calculated by analyzing the signal intensity of the band representing immunoprecipitates detected with the anti-CBF antibody (Figure 3A–C). Together with the nuclear localization data (Figure 2), the results suggest that Trx-h2(G/A) was distributed both in the cytoplasm and nucleus at 22 °C, and interacted with the basal level of CBFs expressed at warm temperature, which are likely involved in the regulation of plant growth and development [38]. Additionally, the cold-induced nuclear translocation rate of Trx-h2(G/A) was faster than that of Trx-h2. These results were verified by the BiFC assay, which was performed using CBF1 as a representative CBF (Figure 3D,E). In the BiFC assay, *Trx-h2-YN* and *Trx-h2(G/A)-YN* were transformed separately into *A. tumefaciens* strain GV3101, and the transformed cells were co-infiltrated into *N. benthamiana* leaves along with the *YC-CBF1* construct, which was compared with the negative controls (Figure S2). To confirm the nuclear localization of Trx-h2 and Trx-h2(G/A), we included the *NLS-RFP* construct as a nuclear marker. While the interaction between Trx-h2 and CBF1 in the nucleus was detected only at 4 °C (Figure 3D), the interaction between Trx-h2(G/A) and CBF1 was detected both at 22 °C and 4 °C. Additionally, the interaction signal between Trx-h2(G/A) and CBF1 was stronger than that between Trx-h2 and CBF1 (Figure 3E). The interaction of Trx-h2 and Trx-h2(G/A) with CBF1 was quantified by counting the number of yellow fluorescent spots under the confocal microscope (Figure 2F).

### 3.4. Trx-h2(G/A) Reduces and Structurally Alters CBF1 at Warm and Cold Temperatures

**Figure 4:**
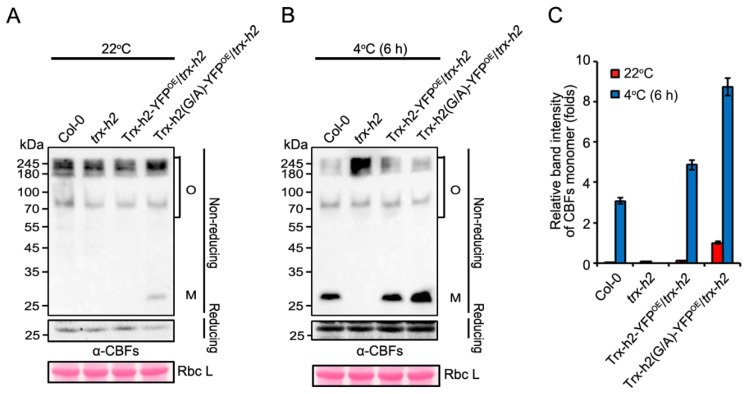
Effect of the Gly^2^ residue of Trx-h2 on the reduction and dissociation of CBF oligomers into monomers at warm (22 °C) and cold (4 °C) temperatures. (**A**,**B**) Cold-induced structural switching of CBFs from the disulfide-bonded oligomeric state to the reduced monomeric CBFs in Col-0, *trx-h2*, Trx-h2-YFP^OE^/*trx-h2*, and Trx-h2(G/A)-YFP^OE^/*trx-h2* plants incubated at 22 °C (**A**) and 4°C (**B**) for 6 h. Proteins were separated on reducing (lower panel) and non-reducing (upper panel) polyacrylamide gels. Structural changes in CBFs were analyzed by Western blot analysis with anti-CBF antibody. Labels O and M on the right side of the Western blot indicate CBF oligomers and monomers, respectively. (**C**) Quantitative analysis of CBF monomers in panels (**A**,**B**) measured by densitometry and quantified by the ImageJ software. The intensity of CBF monomers was normalized first relative to that of RbcL and then relative to the value of Trx-h2(G/A) at 22 °C shown in the last lane in panel (**A**), which was set to 1. Data represent mean ± s.e.m. (*n* = 3 biologically independent samples).

To activate CBFs under cold conditions, the CBF oligomers linked with disulfide bonds must be reduced by the nuclear-translocated Trx-h2, which dissociated their oligomeric structures into monomers [27]. Given that the speed of nuclear translocation and the quantity of translocated Trx-h2(G/A) were greater than those of Trx-h2 at cold temperature (Figure 3), we compared the structural transition rate of CBFs among Col-0, *trx-h2*, Trx-h2-YFP^OE^/*trx-h2*, and Trx-h2(G/A)-YFP^OE^/*trx-h2* plants at 22 °C and 4 °C. The expression of CBFs in the various genotypes showed similar levels analyzed by reducing SDS-PAGE gels (lower panels of Figure 4A,B). Thus, to investigate the structural switching of CBFs, total plant proteins were separated by SDS-PAGE on a non-reducing gel, and subjected to Western blotting with anti-CBF antibody. At 22 °C, a small amount of monomeric CBFs was detected in Trx-h2(G/A)-YFP^OE^/*trx-h2* plants, whereas all the other genotypes exclusively contained oligomeric CBFs (Figure 4A). By contrast, cold induced the reduction in CBFs in Col-0, Trx-h2-YFP^OE^/*trx-h2*, and Trx-h2(G/A)-YFP^OE^/*trx-h2* plants, but not in *trx-h2* mutant plants, confirming that Trx-h2 is essential for the cold-induced reduction and dissociation of CBFs (Figure 4B). A stronger shift in CBF structure from oligomeric to monomeric form was detected in Trx-h2(G/A)-YFP^OE^/*trx-h2* plants at 4 °C for 6 h, in which most of CBF oligomers were dissociated into monomers by cold (Figure 4B). The band intensities of CBF monomer in various genotypes (in non-reducing panels of Figure 4A,B) were measured with a densitometer and quantified by the ImageJ software. Based on the band intensity of CBF monomer in Trx-h2(G/A)-YFP^OE^/*trx-h2* plants incubated at 22 °C (Figure 4A: in the last lane of non-reducing gel) was set to 1, relative band intensities of CBF monomers in various genotypes were compared (Figure 4A,B, non-reducing gel). From the results, it can be concluded that Trx-h2(G/A) reduces CBFs and dissociates their protein structures from oligomer to monomers more efficiently than Trx-h2 under the cold temperatures.

### 3.5. Comparison of Trx-h2 and Trx-h2(G/A) for the CBF1 Activation under the Warm and Cold Temperatures

**Figure 5:**
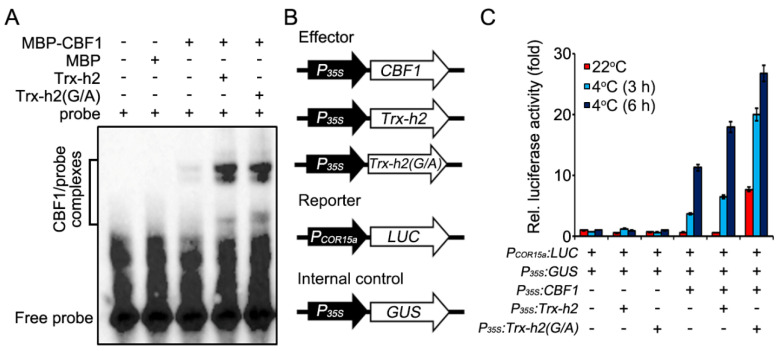
Comparison of the ability of Trx-h2 and Trx-h2(G/A) to activate CBF1 and consequently the *COR15a* promoter-driven *LUC* gene at different temperatures. (**A**) EMSA showing the binding of MBP-CBF1 to the biotin-labeled *COR15a* promoter probe in the presence of NADPH, Trx reductase, and Trx-h2 or Trx-h2(G/A). MBP was used as a negative control. Reaction products were separated on 6% polyacrylamide gels, and DNA–protein complexes were detected by Western blot analysis using anti-biotin. (**B**) Schematic representation of effector, reporter, and internal control constructs used in the LUC assay. (**C**) Quantification of LUC activity in *N. benthamiana* leaves co-expressing *P_35S_:CBF1* with *P_35S_*:*Trx-h2* or *P_35S_:Trx-h2(C/S)* at 22 °C or 4 °C for different durations. Data represent mean ± s.e.m. (*n* = 3 biologically independent samples).

Trx-h2-mediated reduction and structural dissociation of CBFs is necessary for the binding of CBFs to *COR* gene promoters under cold conditions [27]. Therefore, we investigated whether Trx-h2(G/A) can trigger the CBFs to bind to the *COR* gene promoter by performing EMSA. In this experiment, we incubated the MBP-CBF1 recombinant protein or MBP (negative control) with a biotin-labeled probe of *COR15a* promoter in the presence or absence of Trx-h2 and Trx-h2(G/A) proteins expressed in *E. coli*. The reaction mixture was supplemented with NADPH and Trx reductase to facilitate electron transfer from Trx-h2 to CBF1. Comparing with MBP to induce CBF1 to bind to the *COR15a* promoter, both Trx-h2 and Trx-h2(G/A) critically enhanced the ability of CBF1 to bind to the *COR15a* promoter analyzed by in vitro EMSA assay (Figure 5A). Next, we compared the ability of Trx-h2 and Trx-h2(G/A) to induce the transcription of the *COR15a* promoter by CBF1 using the *LUC* reporter gene in vivo (Figure 5B). The *LUC* reporter gene driven by the *COR15a* promoter was expressed in *N. benthamiana* leaves along with the Trx-h2 or Trx-h2(G/A) effector, and the plants were incubated at 22 °C and 4 °C. GUS was used as an internal control, and LUC activity was normalized relative to GUS activity (Figure 5C). The transient expression of *CBF1* slightly increased LUC activity at 4 °C; by contrast, the co-expression of CBF1 with Trx-h2 greatly enhanced LUC activity at 4 °C but not at 22 °C. At 4 °C, LUC activity was much higher in leaves co-expressing CBF1 and Trx-h2(G/A) than in those co-expressing CBF1 and Trx-h2, especially at the early time points. In particular, Trx-h2(G/A) was able to activate CBF1 and consequently induce LUC activity at 22 °C, consistent with the result that a part of Trx-h2(G/A) localized to the nucleus and interacted with CBFs at warm temperature (Figure 2 and Figure 3).

### 3.6. Comparison of the Ability of Trx-h2 and Trx-h2(G/A) to Activate CORs Expression under Cold Stress

**Figure 6:**
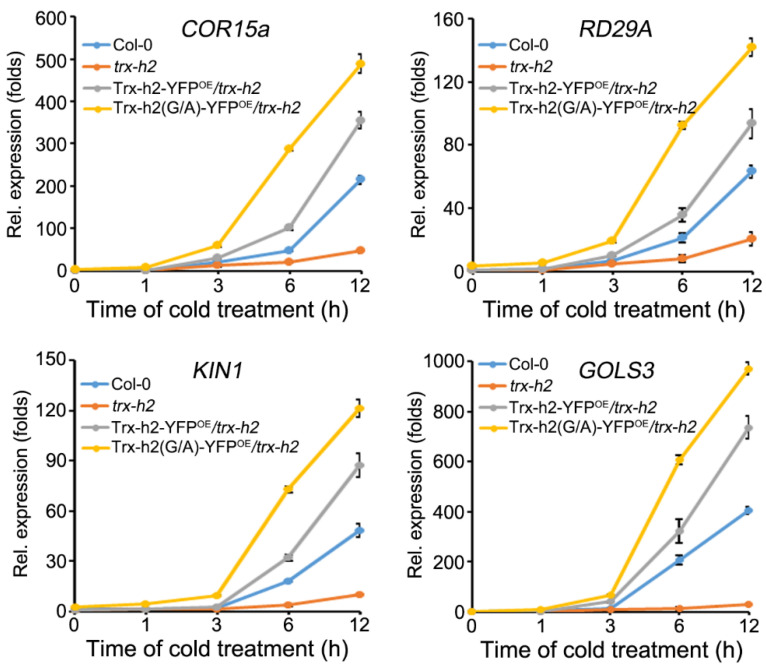
Comparison of the kinetics of Trx-h2- and Trx-h2(G/A)-mediated CORs expression under cold stress. Transcript levels of *COR15a*, *RD29A*, *KIN1*, and *GOLS3* were examined by qRT-PCR in Col-0, *trx-h2*, Trx-h2-YFP^OE^/*trx-h2*, and Trx-h2(G/A)-YFP^OE^/*trx-h2* plants incubated at 4 °C for the indicated duration. Data represent mean ± s.e.m. (*n* = 3 biologically independent samples).

CBF-induced expression of *CORs* is the major defense signaling pathway employed by plants under cold stress [39]. As Trx-h2(G/A) reduced and activated CBFs more efficiently than Trx-h2, we compared the capacity of Trx-h2 and Trx-h2(G/A) to activate *COR* gene expression in Col-0, *trx-h2*, Trx-h2-YFP^OE^/*trx-h2*, and Trx-h2(G/A)-YFP^OE^/*trx-h2* plants. These plants were grown in Murashige and Skoog (MS) medium for 14 days, and then transferred to 4 °C for 0, 1, 3, 6, and 12 h. To analyze the initial response of Trx-h2(G/A) at 4 °C, we examined *COR* gene expression at the early time points (Figure 6). The expression of *COR* genes was highly up-regulated by cold treatment in Col-0, Trx-h2-YFP^OE^/*trx-h2*, and Trx-h2(G/A)-YFP^OE^/*trx-h2* plants compared with *trx-h2* plants. Furthermore, the expression level of *COR* genes in Trx-h2-YFP^OE^/*trx-h2* and Trx-h2(G/A)-YFP^OE^/*trx-h2* plants was much higher than that in Col-0 plants. In particular, the rate of enhancement of *CORs* expression in Trx-h2(G/A)-YFP^OE^/*trx-h2* plants at the early time points was much faster than that in other genotypes. The results suggest that Trx-h2(G/A), previously localized to the nucleus at warm temperatures, can activate CBFs faster than Trx-h2 against cold stress.

### 3.7. Comparison of the Freezing Tolerance of Plants Expressing Trx-h2 and Trx-h2(G/A)

**Figure 7:**
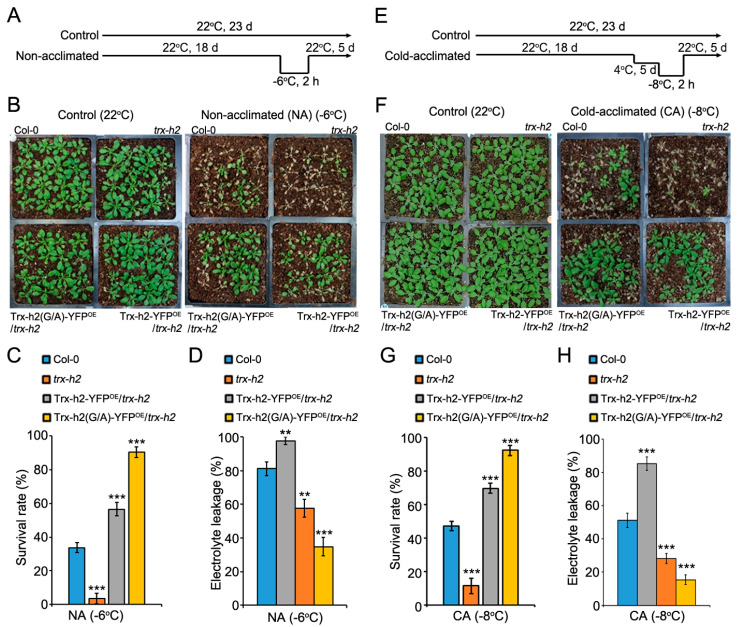
Comparison of the freezing tolerance of the NA and CA plants of Col-0, *trx-h2*, Trx-h2-YFP^OE^/*trx-h2*, and Trx-h2(G/A)-YFP^OE^/*trx-h2* genotypes. (**A**,**E**) Freezing tolerance assay of plants in the NA treatment (**A**) and CA treatment (**E**). In the NA treatment, 18-day-old plants were placed in the freezing chamber at the desired temperature for 2 h. In the CA treatment, plants were pre-incubated at 4 °C for 5 days before the freezing test. After exposing the plants to freezing stress, NA and CA plants were incubated at 22 °C for 5 days for recovery. (**B**–**H**) Evaluation of the freezing stress tolerance of plants based on their recovery (**B**,**F**), survival rate (**C**,**G**), and electrolyte leakage (**D**,**H**). Data represent mean ± s.e.m. (*n* = 3 biologically independent samples for survival rates, and *n* = 10 biologically independent samples for electrolyte leakage). Significant differences are indicated by asterisks (** *p* < 0.01, *** *p* < 0.001; Student’s *t*-test).

As the rate of *COR* gene activation in Trx-h2(G/A)-YFP^OE^/*trx-h2* plants was faster than that in Trx-h2-YFP^OE^/*trx-h2* plants at cold temperature, we investigated the effect of myristoylation of Trx-h2 on freezing tolerance in Arabidopsis *in vivo*. The freezing tolerance of soil-grown Col-0, *trx-h2*, Trx-h2-YFP^OE^/*trx-h2*, and Trx-h2(G/A)-YFP^OE^/*trx-h2* plants was compared between non-acclimated (NA) and cold-acclimated (CA) treatments. In the NA treatment, plants grown at optimal temperature for 18 days were directly transferred to a freezing chamber, which was cooled down to the target freezing temperature. Plants were incubated at the target temperature for 2 h and then transferred to the growth chamber at 22 °C (Figure 7A). Freezing tolerance levels of plants were evaluated by monitoring their phenotypic recovery (Figure 7B), survival rate (Figure 7C), and electrolyte leakage (%) (Figure 7D). Electrolyte leakage caused by freezing stress-mediated damage to the plasma membrane is a strong indicator of the freezing tolerance of plants [40]. The *trx-h2* plants were highly sensitive to freezing stress compared with other genotypes, which strongly suggests that Trx-h2 is essential for conferring freezing tolerance to plants. In addition, Trx-h2(G/A)-YFP^OE^/*trx-h2* plants showed slightly higher freezing tolerance than Trx-h2-YFP^OE^/*trx-h2* plants (Figure 7B), which is in agreement with the results of *CORs* expression (Figure 6). These results were consistent with the survival rate and electrolyte leakage (%) of various genotypes. In addition, to analyze the effect of cold acclimation (CA) on freezing tolerance, we exposed the plants to low temperature (4 °C) for 5 days before subjecting them to freezing stress (Figure 7E). Similar to plants in the NA treatment, the freezing tolerance of *trx-h2* plants in the CA treatment also showed a highly sensitive phenotype against freezing stress, confirming that Trx-h2 is necessary for freezing tolerance in plants (Figure 7F). Furthermore, Trx-h2(G/A)-YFP^OE^/*trx-h2* plants displayed slightly higher freezing tolerance than Col-0 and Trx-h2-YFP^OE^/*trx-h2* plants (Figure 7F–H). These results suggest that a part of Trx-h2(G/A) previously localized to the nucleus in Trx-h2(G/A)-YFP^OE^/*trx-h2* plants under normal conditions rapidly activates CBFs, to protect plants from a cold snap.

## 4. Discussion

We previously reported that Trx-h2 plays a critical role in the redox-dependent regulation of the structure and activity of CBFs under cold stress conditions [27]. During the cold signaling in plants, cold shock triggers the demyristoylation of the Gly^2^ residue of Trx-h2, and demyristoylated Trx-h2 is rapidly translocated from the cytoplasm to the nucleus, followed by the activation of CBFs. In this study, we investigated the significance of the myristoylation of Trx-h2 in the freezing tolerance of plants by analyzing the biochemical and physiological properties of the recombinant Trx-h2(G/A) protein and of Trx-h2(G/A)-YFP^OE^/*trx-h2* transgenic plants.

Several studies have shown that the myristoylated state of specific proteins critically affects the environmental stress resistance of plants. For instance, myristoylation of the plasma membrane-localized clade-E growth-regulating 2 (EGR2) phosphatase is required for freezing tolerance in plants [36]. At warm temperatures, the myristoylated EGR2 phosphatase interacts with OST1 kinase and inhibits its activity. However, under cold stress conditions, the accumulation of demyristoylated EGR2 disrupts the EGR2–OST1 interaction, leading to the activation of OST1 kinase and enhancing the freezing tolerance of plants. Additionally, myristoylation of the salt overly sensitive3 (SOS3) protein is important for the salt stress resistance in plants. Thus, plants expressing the SOS3(G/A) mutant protein, which cannot be myristoylated, exhibit a highly salt-sensitive phenotype [37]. In addition, myristoylation regulates abscisic acid (ABA) signaling in plants. Meanwhile, the plant hormone ABA inhibits myristoylation and enhances nuclear translocation of RING domain ligase 1 (RGLG1)-E3 ligase. The result promotes the formation of the RGLG1–receptor–phosphatase complex, and enhances the ABA response and salt or osmotic stress [41].

**Figure 8:**
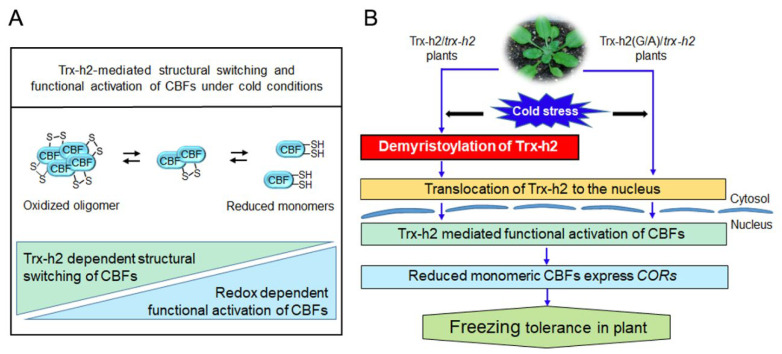
Proposed model demonstrating the significance of the myristyl group attached to the Gly^2^ residue of Trx-h2 in the structural switching and activation of CBF1 and, consequently, the regulation of plant freezing tolerance. (**A**) Under cold conditions, Trx-h2 induces the structural switching of CBFs from the oxidized oligomeric state to the reduced monomeric state. The reduced CBF monomers then bind to *COR* promoters and activate their gene expression, which enhances plant cold tolerance. (**B**) When plants are exposed to cold stress, the Gly^2^ residue of Trx-h2 is cleaved, and the demyristoylated Trx-h2 is translocated to the nucleus, where it interacts with CBFs. In the nucleus, Trx-h2 reduces and activates CBFs to transcribe *CORs*. Unlike Trx-h2, Trx-h2(G/A) is partially localized at the nucleus at warm temperature, and can rapidly respond to a cold snap. Therefore, under cold conditions, the rate of CBF activation and *COR* gene expression is faster in Trx-h2(G/A)-YFP^OE^/*trx-h2* plants than in plants expressing *Trx-h2*. The rapid and efficient response of Trx-h2(G/A)-YFP^OE^/*trx-h2* plants to cold shock results in enhanced freezing tolerance.

Similar to other proteins, the subcellular localization of Trx-h2 is determined by its myristoylation or demyristoylation status, which in turn decides the activity of cold-responsive CBFs (Figure 8). In the nucleus, Trx-h2 reduces the disulfide-bonded inactive CBF oligomers to dissociate them into active monomers. Then, the reduced monomeric CBFs activate the expression of *COR* genes to confer freezing tolerance to plants. Therefore, when we traced the subcellular localization of Trx-h2(G/A), a mutant protein incapable of myristoylation, a part of the Trx-h2(G/A) protein was localized to the nucleus at warm temperatures and was therefore ready for the reduction and activation of CBFs upon a cold snap. Thus, in comparison with plants expressing *Trx-h2*, the Trx-h2(G/A)-YFP^OE^/*trx-h2* plants exhibited more efficient and rapid response to freezing stress.

In addition to Trx-h2, several other Arabidopsis Trx-h isoforms are modified by the conjugation of specific fatty acid moieties to their N-terminal amino acids [35,42]. In contrast to myristorylation of cellular proteins, it should be demyristorylated to reversibly transduce the extracellular signals into downstream signaling cascades. In fact, the enzymes involved in protein demyristoylation have been identified in human and bacteria, whereas the N-myristoylation of proteins was reported to be an irreversible process in plants, until now [43,44]. Therefore, if we identify the demyristoylase enzyme from plant sources, it might be highly important to ascertain the regulation mechanism on the diverse physiological processes in plants, including growth and development [45,46]. In addition, considering the results of the current study, the *Trx-h2(G/A)* mutant allele could potentially be used for the production of highly cold resistant crops that exhibit high productivity, especially under cold conditions.

## Data Availability

Data is contained within the article and Supplementary Material.

## Supplementary Materials

The following are available online at https://www.mdpi.com/2076-3921/10/8/1287/s1: Figure S1: Generation of transgenic Arabidopsis lines in the *trx-h2* mutant background overexpressing Trx-h2 and its point mutation variant Trx-h2(G/A) (Trx-h2-YFP^OE^/*trx-h2* and Trx-h2(G/A)-YFP^OE^/*trx-h2*, respectively); Figure S2: Interaction of Trx-h2 with YFP as negative controls in BiFC assay; Table S1: List of primers used in this study.

## References

[1] Mittler R. (2006). Abiotic stress, the field environment and stress combination. Trends Plant Sci..

[2] Apel K., Hirt H. (2004). Reactive Oxygen Species: Metabolism, Oxidative Stress, and Signal Transduction. Annu. Rev. Plant Biol..

[3] Choudhury S., Panda P., Sahoo L., Panda S.K. (2013). Reactive oxygen species signaling in plants under abiotic stress. Plant Signal. Behav..

[4] Waszczak C., Carmody M., Kangasjärvi J. (2018). Reactive Oxygen Species in Plant Signaling. Annu. Rev. Plant Biol..

[5] Zandalinas S.I., Fichman Y., Devireddy A.R., Sengupta S., Azad R.K., Mittler R. (2020). Systemic signaling during abiotic stress combination in plants. Proc. Natl. Acad. Sci. USA.

[6] Foyer C.H., Noctor G. (2013). Redox Signaling in Plants. Antioxid. Redox Signal..

[7] Mittler R., Vanderauwera S., Suzuki N., Miller G., Tognetti V.B., Vandepoele K., Gollery M., Shulaev V., Van Breusegem F. (2011). ROS signaling: The new wave?. Trends Plant Sci..

[8] Huang H., Ullah F., Zhou D.-X., Yi M., Zhao Y. (2019). Mechanisms of ROS Regulation of Plant Development and Stress Responses. Front. Plant Sci..

[9] Castro B., Citterico M., Kimura S., Stevens D.M., Wrzaczek M., Coaker G. (2021). Stress-Induced Reactive Oxygen Species Compartmentalization, Perception and Signalling. Nat. Plants.

[10] Mittler R., Vanderauwera S., Gollery M., Van Breusegem F. (2004). Reactive oxygen gene network of plants. Trends Plant Sci..

[11] Lee E.S., Kang C.H., Park J.H., Lee S.Y., Lee M.E.S., Park M.J.H. (2018). Physiological Significance of Plant Peroxiredoxins and the Structure-Related and Multifunctional Biochemistry of Peroxiredoxin 1. Antioxid. Redox Signal..

[12] Jang H.H., Lee K.O., Chi Y.H., Jung B.G., Park S.K., Park J.H., Lee J.R., Lee S.S., Moon J.C., Yun J.W. (2004). Two Enzymes in One: Two Yeast Peroxiredoxins Display Oxidative Stress-Dependent Switching from a Peroxidase to a Molecular Chaperone Function. Cell.

[13] Chae H.B., Kim M.G., Kang C.H., Park J.H., Lee E.S., Lee S.-U., Chi Y.H., Paeng S.K., Bin Bae S., Wi S.D. (2021). Redox sensor QSOX1 regulates plant immunity by targeting GSNOR to modulate ROS generation. Mol. Plant.

[14] Sun L., Ren H., Liu R., Li B., Wu T., Sun F., Liu H., Wang X., Dong H. (2010). An h-Type Thioredoxin Functions in Tobacco Defense Responses to Two Species of Viruses and an Abiotic Oxidative Stress. Mol. Plant Microbe Interact..

[15] Li Y.-C., Ren J.-P., Cho M.-J., Zhou S.-M., Kim Y.-B., Guo H.-X., Wong J.H., Niu H.-B., Kim H.-K., Morigasaki S. (2009). The Level of Expression of Thioredoxin is Linked to Fundamental Properties and Applications of Wheat Seeds. Mol. Plant.

[16] Meng L., Wong J.H., Feldman L.J., Lemaux P.G., Buchanan B.B. (2010). A membrane-associated thioredoxin required for plant growth moves from cell to cell, suggestive of a role in intercellular communication. Proc. Natl. Acad. Sci. USA.

[17] Park S.K., Jung Y.J., Lee J.R., Lee Y.M., Jang H.H., Lee S.S., Park J.H., Kim S.Y., Moon J.C., Lee S.Y. (2009). Heat-Shock and Redox-Dependent Functional Switching of an h-Type Arabidopsis Thioredoxin from a Disulfide Reductase to a Molecular Chaperone. Plant Physiol..

[18] Tada Y., Spoel S., Pajerowska-Mukhtar K., Mou Z., Song J., Wang C., Zuo J., Dong X. (2008). Plant Immunity Requires Conformational Charges of NPR1 via S-Nitrosylation and Thioredoxins. Science.

[19] Hemsley P.A. (2014). The importance of lipid modified proteins in plants. N. Phytol..

[20] Friso G., Van Wijk K.J. (2015). Update: Post-Translational Protein Modifications in Plant Metabolism. Plant Physiol..

[21] He Z., Huang T., Ao K., Yan X., Huang Y. (2017). Sumoylation, Phosphorylation, and Acetylation Fine-Tune the Turnover of Plant Immunity Components Mediated by Ubiquitination. Front. Plant Sci..

[22] Park J.H., Kang C.H., Nawkar G.M., Lee E.S., Paeng S.K., Chae H.B., Chi Y.H., Kim W.Y., Yun D.-J., Lee S.Y. (2018). EMR, a cytosolic-abundant ring finger E3 ligase, mediates ER-associated protein degradation in Arabidopsis. N. Phytol..

[23] Karve T.M., Cheema A.K. (2011). Small Changes Huge Impact: The Role of Protein Posttranslational Modifications in Cellular Homeostasis and Disease. J. Amino Acids.

[24] Kim M.-Y., Bae J.-S., Kim T.-H., Park J.-M., Ahn Y.H. (2011). Role of Transcription Factor Modifications in the Pathogenesis of Insulin Resistance. Exp. Diabetes Res..

[25] Martin D.D., Beauchamp E., Berthiaume L.G. (2011). Post-Translational Myristoylation: Fat Matters in Cellular Life and Death. Biochimie.

[26] Jiang H., Zhang X., Chen X., Aramsangtienchai P., Tong Z., Lin H. (2017). Protein Lipidation: Occurrence, Mechanisms, Biological Functions, and Enabling Technologies. Chem. Rev..

[27] Lee E.S., Park J.H., Wi S.D., Kang C.H., Chi Y.H., Chae H.B., Paeng S.K., Ji M.G., Kim W.-Y., Kim M.G. (2021). Redox-dependent structural switch and CBF activation confer freezing tolerance in plants. Nat. Plants.

[28] Clough S.J., Bent A. (1998). Floral dip: A simplified method forAgrobacterium-mediated transformation ofArabidopsis thaliana. Plant J..

[29] Nawkar G.M., Kang C.H., Maibam P., Park J.H., Jung Y.J., Chae H.B., Chi Y.H., Jung I.J., Kim W.Y., Yun D.-J. (2017). HY5, a positive regulator of light signaling, negatively controls the unfolded protein response inArabidopsis. Proc. Natl. Acad. Sci. USA.

[30] Zhang L., Zhang L., Xia C., Gao L., Hao C., Zhao G., Jia J., Kong X. (2017). A Novel Wheat C-bZIP Gene, TabZIP14-B, Participates in Salt and Freezing Tolerance in Transgenic Plants. Front. Plant Sci..

[31] Kang C.H., Lee Y.M., Park J.H., Nawkar G.M., Oh H.T., Kim M.G., Lee S.I., Kim W.Y., Yun D., Lee S.Y. (2016). Ribosomal P3 protein AtP3B ofArabidopsisacts as both protein and RNA chaperone to increase tolerance of heat and cold stresses. Plant Cell Environ..

[32] Frottin F., Martinez A., Peynot P., Mitra S., Holz R.C., Giglione C., Meinnel T. (2006). The Proteomics of N-terminal Methionine Cleavage. Mol. Cell. Proteom..

[33] Martinez A., Traverso J.A., Valot B., Ferro M., Espagne C., Ephritikhine G., Zivy M., Giglione C., Meinnel T. (2008). Extent of N-terminal modifications in cytosolic proteins from eukaryotes. Proteomics.

[34] Traverso J.A., Micalella C., Martinez A., Brown S.C., Satiat-Jeunemaitre B., Meinnel T., Giglione C. (2013). Roles of N-Terminal Fatty Acid Acylations in Membrane Compartment Partitioning: Arabidopsis h-Type Thioredoxins as a Case Study. Plant Cell.

[35] Hantschel O., Nagar B., Guettler S., Kretzschmar J., Dorey K., Kuriyan J., Superti-Furga G. (2003). A Myristoyl/Phosphotyrosine Switch Regulates c-Abl. Cell.

[36] Ding Y., Lv J., Shi Y., Gao J., Hua J., Song C., Gong Z., Yang S. (2018). EGR 2 phosphatase regulates OST 1 kinase activity and freezing tolerance in Arabidopsis. EMBO J..

[37] Ishitani M., Liu J., Halfter U., Kim C.-S., Shi W., Zhu J.-K. (2000). SOS3 Function in Plant Salt Tolerance Requires N-Myristoylation and Calcium Binding. Plant Cell.

[38] Zhu J.-K. (2016). Abiotic Stress Signaling and Responses in Plants. Cell.

[39] Shi Y., Ding Y., Yang S. (2018). Molecular Regulation of CBF Signaling in Cold Acclimation. Trends Plant Sci..

[40] Park J., Lim C.J., Shen M., Park H.J., Cha J.-Y., Iniesto E., Rubio V., Mengiste T., Zhu J.-K., Bressan R.A. (2018). Epigenetic switch from repressive to permissive chromatin in response to cold stress. Proc. Natl. Acad. Sci. USA.

[41] Belda-Palazon B., Julian J., Coego A., Wu Q., Zhang X., Batistic O., AlQuraishi S.A., Kudla J., An C., Rodriguez P.L. (2019). ABA inhibits myristoylation and induces shuttling of the RGLG 1 E3 ligase to promote nuclear degradation of PP 2 CA. Plant J..

[42] Resh M.D. (2006). Trafficking and signaling by fatty-acylated and prenylated proteins. Nat. Chem. Biol..

[43] Burnaevskiy N., Fox T.G., Plymire D.A., Ertelt J.M., Weigele B.A., Selyunin A.S., Way S.S., Patrie S.M., Alto N.M. (2013). Proteolytic Elimination of N-Myristoyl Modifications by the Shigella Virulence Factor IpaJ. Nat. Cell Biol..

[44] Burnaevskiy N., Peng T., Reddick L.E., Hang H.C., Alto N.M. (2015). Myristoylome Profiling Reveals a Concerted Mechanism of ARF GTPase Deacylation by the Bacterial Protease IpaJ. Mol. Cell.

[45] Hashiguchi A., Komatsu S. (2016). Impact of Post-Translational Modifications of Crop Proteins under Abiotic Stress. Proteomes.

[46] Su M.-G., Weng J.T.-Y., Hsu J.B.-K., Huang K.-Y., Chi Y.-H., Lee T.-Y. (2017). Investigation and identification of functional post-translational modification sites associated with drug binding and protein-protein interactions. BMC Syst. Biol..

