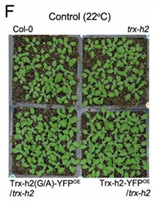# Correction: Lee et al. Demyristoylation of the Cytoplasmic Redox Protein Trx-h2 Is Critical for Inducing a Rapid Cold Stress Response in Plants. *Antioxidants* 2021, *10*, 1287

**DOI:** 10.3390/antiox11112223

**Published:** 2022-11-11

**Authors:** Eun Seon Lee, Joung Hun Park, Seong Dong Wi, Ho Byoung Chae, Seol Ki Paeng, Su Bin Bae, Kieu Anh Thi Phan, Min Gab Kim, Sang-Soo Kwak, Woe-Yeon Kim, Dae-Jin Yun, Sang Yeol Lee

**Affiliations:** 1 Division of Applied Life Science (BK21+) and PMBBRC, Gyeongsang National University, Jinju 52828, Korea; 2 College of Pharmacy, Gyeongsang National University, Jinju 52828, Korea; 3 Plant Systems Engineering Research Center, KRIBB, Daejeon 34141, Korea; 4 Department of Biomedical Science & Engineering, Konkuk University, Seoul 05029, Korea

## Error in Figure

In the original publication [1], there was a mistake in the picture of ‘*Arabidopsis* control, cultured at 22 °C (Figure 7F)’ as published. Because, at the final step of paper preparation, the first author of this paper, Dr. (Mrs.) Lee, E.S., had given birth to a baby and had a maternity leave, the other co-authors finalized the arrangement of the figures, but made a mistake in the selection process of the correct figures from the numbers of her laboratory notebooks. ‘The mistake happened in the selection of right figure is corrected at this time’. The original and corrected pictures of ‘*Arabidopsis* control, cultured at 22 °C (Figure 7F)’ appear below. The authors state that the scientific conclusions are unaffected. This correction was approved by the Academic Editor. The original publication has also been updated.

Original Figure 7F:


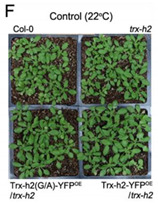


Corrected Figure 7F: