# Supplementary material for: Demyristoylation of the Cytoplasmic Redox Protein Trx-h2 Is Critical for Inducing a Rapid Cold Stress Response in Plants
Source: Antioxidants (Basel). 2021 Aug 13;10(8):1287. doi: 10.3390/antiox10081287 (PMC8389195; doi:10.3390/antiox10081287)
Supplement: Supplementary file 1 [file antioxidants-10-01287-s001.zip › antioxidants-1316203-supplementary.pdf]

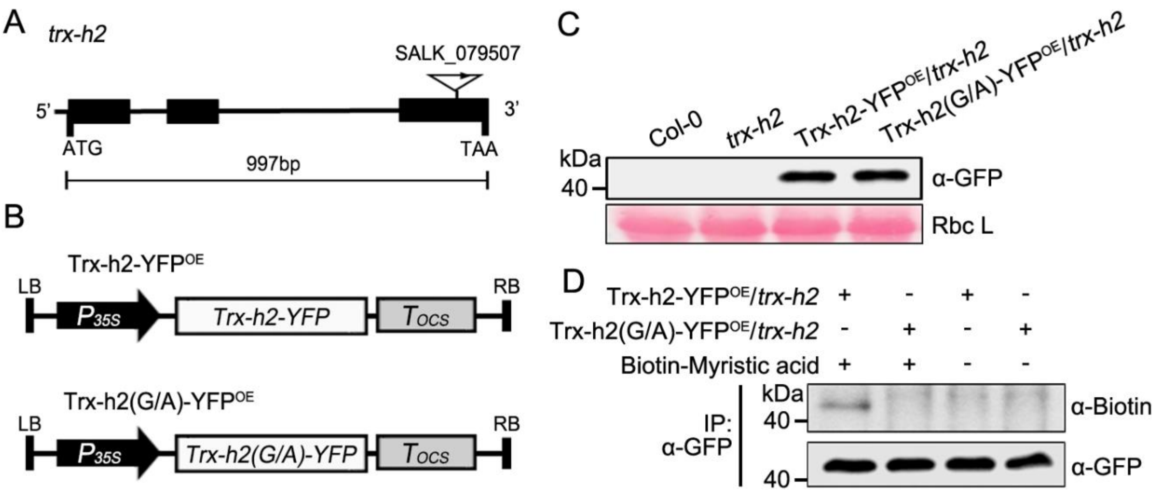

Figure S1. Generation of transgenic lines expressing Trx-h2 and its point-mutant variant, Trx-h2(G/A), in *trx-h2* background, resulting in Trx-h2-YFP^{OE}/*trx-h2* and Table 2. G/A)-YFP^{OE}/*trx-h2* plants. **A**. A schematic diagram of *trx-h2* knock-out genomic structure (SALK_079507). T-DNA is inserted at the third exon of Trx-h2. **B**. Schematic diagrams of DNA constructs for the generation of overexpression lines of YFP-tag fusions of Trx-h2 and Trx-h2(G/A). The genes are expressed under the control of the CaMV *35S* promoter and octopine synthase (OCS) terminator. **C**. Expression levels of Trx-h2 protein in the transgenic Arabidopsis analyzed by western blot with anti-GFP antibody. Rbc L stained with Ponceau S was used as loading controls. **D**. Biotin-labeled Trx-h2-myristate was examined from the plants of Trx-h2-YFP^{OE}/*trx-h2* and Trx-h2(G/A)-YFP^{OE}/*trx-h2* by western blotting.

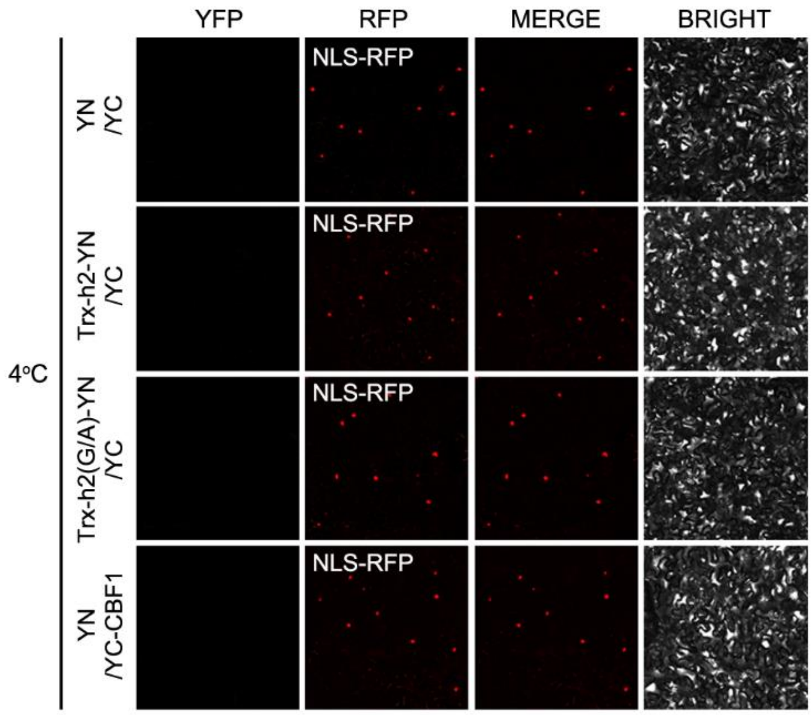

Figure S2. Interaction of Trx-h2 with YFP as negative controls in BiFC assay. BiFC assay performed by co-expressing *YN* and *YC vectors, Trx-h2-YN* or *Trx-h2(G/A)-YN* and *YC vector,* and *YN vector and YC-CBF1* in *N. benthamiana* leaves. Samples were incubated at 4°C for 6 h. YFP-signals analyzed under the confocal microscopy were merged with that of NLS-RFP used as a nuclear marker (Merge).

Table S1. List of primers used in this study.

Accession Number	Primer name	Primer sequence (5'→3')
	Plasmid construction	
At5G39950	Trx-h2 F	ATCGATATGGGAGGAGCTTTATCAACT
	Trx-h2 R	TCTAGATGCTCTGAGTTTGCTAACTTTCTT
	Trx-h2 attB1 F	AAAAAGCAGGCCATATGGGAGGAGCTTTATCAAC
	Trx-h2 attB2 R	AGAAAGCTGGGTTATGCTCTGAGTTTGCTAA
	Trx-h2(G/A) F	ATCGATATGGCAGGAGCTTTATCAAC
	Trx-h2(G/A) attB1 F	AAAAAGCAGGCCATATGGCAGGAGCTTTATCAAC
At4G25490	CBF1 F	GAATTCATGAACTCATTTTCAGCT
	CBF1 R	AAGCTTTTAGTAACTCCAAAGCGA
	CBF1 attB1 F	AAAAAGCAGGCCATATGAACTCATTTTCAG
	CBF1 attB1 R	AGAAAGCTGGGTTAGTAACTCCAAAGCG
	attB1	GGGGACAAGTTTGTACAAAAAAGCAGGCCAT
	attB2	GGGGACCACTTTGTACAAGAAAGCTGGGT
	EMSA assay	
	CRT/DRE F	ATTTCATGGCCGACCTGCTTTTT
	CRT/DRE R	AAAAAGCAGGTCGGCCATGAAAT
	Quantitative real-time PCR (qRT-PCR)	
At4G05320	UBQ10_qF	GGCCTTGTATAATCCCTGATGAATAAG
	UBQ10_qR	AAAGAGATAACAGGAACGGAAACATAGT
AT3G18780	ACT2_qF	CTTGCACCAAGCAGCATGAA
	ACT2_qR	CCGATCCAGACACTGTACTTCCTT
AT2G42450	COR15a_qF	AACGAGGCCACAAAGAAAGC
	COR15a_qR	CAGCTTCTTTACCCAATGTATCTGC-
AT5G52310	RD29A_qF	GAAAGGAGGAGGAGGAATGG
	RD29A _qR	AACCAGCCAGATGATTTTGG
AT5G15960	KIN1_qF	TGGAGCTGGAGCACAACA
	KIN1_qR	GACCCGAATCGCTACTTGTTC
AT1G09350	GOLS3_qF	GGAGTGGTTGGTCTGGCTAA
	GOLS3_qR	TTGGTTATCCGGTGGGTAAA
